# NAD+ Biosynthesis Impairment and Acute Kidney Injury after Major Vascular Surgery

**DOI:** 10.3390/antiox12040821

**Published:** 2023-03-28

**Authors:** Annmarie I. Mede, Ginger L. Milne, Dawei Wei, Derek K. Smith, Loren E. Smith

**Affiliations:** 1 Vanderbilt University School of Medicine, Nashville, TN 37232, USA; 2 Departments of Medicine, Vanderbilt University Medical Center, Nashville, TN 37232, USA; 3 Departments of Pharmacology, Vanderbilt University Medical Center, Nashville, TN 37232, USA; 4 Departments of Biostatistics, Vanderbilt University Medical Center, Nashville, TN 37232, USA; 5 Departments of Anesthesiology, Vanderbilt University Medical Center, Nashville, TN 37232, USA

**Keywords:** AKI, kidney injury, NAD, vascular surgery, quinolinate

## Abstract

Acute kidney injury (AKI) is a serious complication after vascular surgery. Reduced synthesis of nicotinamide adenine dinucleotide (NAD+) from tryptophan is associated with an increased risk of AKI in critically ill patients, patients hospitalized with COVID-19, and cardiac surgery patients, and is marked by elevated urinary quinolinate and quinolinate to tryptophan ratios. We measured quinolinate concentrations in vascular surgery patients to determine if impaired NAD+ synthesis was associated with AKI in this patient population. Eight preoperative and eight postoperative vascular surgery patients who developed AKI were selected from a parent study to participate in this single-center case-control study. They were matched with controls who did not develop AKI based on age, sex, BMI, eGFR, hypertension, and diabetes. Urinary quinolinate and tryptophan concentrations were measured at anesthetic induction and on postoperative day one. Two-sided Mann–Whitney U tests were used to compare quinolinate and quinolinate to tryptophan ratios. Multivariate linear regression modeling was used to estimate the relationship between quinolinate and serum creatinine. There was no difference in preoperative or postoperative urine quinolinate concentrations or the preoperative quinolinate to tryptophan ratio between patients that did and did not develop AKI (*p* = 0.07, 0.50, and 0.32, respectively). However, postoperative quinolinate to tryptophan ratios were higher in AKI patients (*p* = 0.04). Further, after adjustment for AKI risk factors, higher preoperative quinolinate concentrations and higher postoperative quinolinate to tryptophan ratios were associated with greater postoperative creatinine increases (*p* = 0.04 and 0.04, respectively). These data suggest that impaired NAD+ synthesis may contribute to AKI development in vascular surgery patients.

## 1. Introduction

Over 350,000 major vascular surgical procedures are performed in the United States annually, with similar per capita rates in other developed nations. Vascular disease and the need for vascular surgery are projected to increase over the next few decades due to the projected growth of the elderly population in many developed nations. Acute kidney injury (AKI) affects up to 40% of patients after major vascular surgery, and is associated with increased perioperative morbidity and mortality [1,2,3,4,5,6]. Vascular surgery patients who develop AKI have increased short-term and long-term mortality, increased risk of the development of or advancement of existing chronic kidney disease, longer hospital stays, and higher rates of perioperative complications, including surgical infections, cardiovascular events, and the need for mechanical ventilation [1,2,3,4,6]. Prevention strategies, such as hemodynamic optimization and minimizing contrast exposure, are limited, and many pharmacologic treatments, including N-acetylcysteine, dopamine, and fenoldopam, have not demonstrated efficacy [7]. No specific AKI treatments are currently available, contributing to the over four-fold increase in mortality suffered by patients that develop AKI during their hospitalization [3].

Nicotinamide adenine dinucleotide is a coenzyme found in all living cells and is present in an oxidized form (NAD+) and a reduced form (NADH). NAD+ serves as an electron carrier for redox reactions in both the mitochondria and cytosol and is essential to many cellular functions, including oxidative phosphorylation and ATP generation, glycolysis, and DNA repair [8,9,10]. NADPH can be generated from NAD+ through phosphorylation of NAD+ by NAD kinases and subsequent conversion by nicotinamide-nucleotide transhydrogenase. In humans, NAD+ can be synthesized de novo from tryptophan, and can also be generated from nicotinamide (NAM), nicotinamide riboside, nicotinamide mononucleotide, and niacin (nicotinic acid) [8,9,10,11].

Physiological stress caused by surgery and acute illness increases the body’s demand for NAD+ and ATP and can also lead to the increased activation of NAD+-consuming enzymes such as poly-ADP-ribose polymerases. During these times of increased need for and utilization of NAD+, impairments in any of the multiple NAD+ synthesis and salvage pathways may predispose an individual to postoperative and critical illness-associated organ dysfunction. Considering that the kidney utilizes more oxygen than any other organ in the body except the heart, and that renal tubule cells are densely packed with mitochondria to support baseline metabolic needs, the kidney may be particularly susceptible to energy depletion and subsequent damage during periods of increased NAD+ consumption and reduced production [9,10,12,13,14]. Indeed, depletion of renal NAM, NAD+, and ATP is observed in patients suffering from AKI [15,16]. Further, animal models of AKI have demonstrated that impaired NAD+ generation worsens kidney injury, and conversely, that enhanced NAD+ synthesis through tryptophan or NAM supplementation diminishes renal injury in models of ischemia-induced and nephrotoxin-induced kidney injury [8,10,11,12,15].

Quinolinate phosphoribosyltransferase (QPRT) is a rate-limiting enzyme in the NAD+ de novo synthesis pathway from tryptophan. QPRT has been shown to be highly expressed in the kidneys of mice, rats, and humans [14]. QPRT activity is crucial to maintaining adequate NAD+ stores in the kidney. In mice, renal ischemia and reperfusion injury downregulates renal expression of QPRT. Conversely, experimental conditions which reduce QPRT activity have been shown to enhance AKI in mouse models [11]. Further, in vitro induction of endoplasmic reticulum stress by multiple different cellular mechanisms in renal proximal tubule cells reduces intracellular NAD+ and quinolinate concentrations, and is associated with QPRT suppression, supporting a role for endoplasmic reticulum stress in the development of impaired NAD+ biosynthesis during AKI [14].

In humans, elevated urinary quinolinate and quinolinate to tryptophan ratios are markers of reduced QPRT activity and impaired de novo NAD+ synthesis from tryptophan [11,14]. In one study of critically ill patients, those who developed postoperative AKI exhibited elevated urinary quinolinate and quinolinate to tryptophan ratios, indicating that impaired de novo NAD+ synthesis may contribute to the development of AKI in critically ill humans and can be noninvasively monitored via the urine [11]. In this study, elevated urinary quinolinate and quinolinate to tryptophan ratios were found to be independent risk factors for AKI and for severe AKI requiring dialysis. In patients hospitalized with the COVID-19 virus, urinary quinolinate to tryptophan ratios have also been shown to be independently associated with the risk of developing AKI [17]. Furthermore, it has been shown that patients who developed AKI after cardiac surgery using cardiopulmonary bypass have higher urinary quinolinate to tryptophan ratios postoperatively [11]. Additionally, these patients’ postoperative quinolinate to tryptophan ratios correlated with the severity of their AKI, suggesting that postoperative urinary quinolinate to tryptophan ratios could be an early biomarker of postoperative AKI in this patient population [14].

While impaired NAD+ synthesis has been implicated in AKI development in cardiac surgery patients exposed to cardiopulmonary bypass, it is unclear if the same pathways play a role in the development of postoperative AKI in other high-risk patient populations, such as vascular surgery patients. Vascular surgery patients have unique risk factors for AKI, including frequent exposure to high volumes of iodinated contrast material and extended periods of renal ischemia due to aortic occlusion and endovascular graft and graft branch positioning. In order to determine if impaired NAD+ synthesis was associated with an increased risk of AKI after major elective vascular surgery, we quantified the urinary quinolinate and tryptophan concentrations in patients’ urine before and on the morning after major vascular surgery to determine if impaired NAD+ synthesis was also associated with a higher risk of AKI after major vascular surgery.

## 2. Materials and Methods

### 2.1. Patient Selection

We tested our hypothesis in a case-control study nested within an ongoing study of postoperative renal function in adults undergoing non-emergent surgery on the major blood vessels of the body at a tertiary academic medical center from 2018 to 2021. Adult vascular surgery patients at high risk for postoperative AKI, defined as having a preoperative estimated glomerular filtration rate (eGFR) < 60 mL/min/1.73 m^2^, were recruited. All patients were taking a statin on a long-term basis before surgery, which is a common medication in this patient population and in some, but not all, studies has been associated with reduced AKI after major vascular surgery [18,19,20,21,22]. Patients on renal replacement therapy (dialysis), patients having undergone renal transplantation, and pregnant patients were excluded from the study. From participants in this ongoing parent study, which included patients undergoing major cardiac and major vascular surgery, vascular surgery patients who developed AKI were identified. At the time of initiation of this case-control study, nine vascular surgery patients enrolled in the parent trial had developed postoperative AKI. Of these nine patients, one patient was missing a preoperative urine sample, and one was missing a postoperative urine sample. The eight vascular surgery patients that developed postoperative AKI and had an available preoperative urine sample were matched to eight vascular surgery patients that did not develop postoperative AKI based on age, sex, body mass index (BMI), baseline eGFR, and past medical history of hypertension and type II diabetes. Similarly, the eight vascular surgery patients with available postoperative urine samples who developed postoperative AKI were matched to eight patients who did not develop postoperative AKI, matched on age, sex, BMI, baseline eGFR, and past medical history of hypertension and type II diabetes. AKI was defined using the Kidney Disease Improving Global Outcomes (KDIGO) serum creatinine criteria, excluding urinary output criteria, due to the low sensitivity of urinary output criteria for AKI during the perioperative period [23]. This study was conducted according to the standards of the Declaration of Helsinki after approval by our Institutional Review Board for Research on Human Subjects. All participants provided written informed consent. Anesthetic and surgical care was provided according to institutional standards, as was preoperative and postoperative care and monitoring.

### 2.2. Data and Sample Collection

Trained research staff collected all patient demographic and clinical data and recorded them in a dedicated Redcap database [24,25]. Patient sex was collected as recorded in the patient’s medical record. Serum creatinine concentration was measured in a Clinical Laboratory Improvement Amendment (CLIA)-certified laboratory prior to surgery and on the mornings of postoperative days one and two. Urine samples were collected at anesthetic induction and on the morning of postoperative day one. Samples were spun at 3000× *g* for 10 min and supernatant was frozen at −80 °C until analyzed for this study. Samples were not exposed to any freeze–thaw cycles before measurements were performed for this study and were thawed immediately before analysis.

### 2.3. Analytical Measurements

To quantify quinolinate and tryptophan concentrations in the urine of our patients, we used a ultraperformance liquid chromatography-tandem mass spectrometry (UPLC-MS/MS) method, as previously described [26]. Briefly, 5 μL of urine was diluted with 45 μL of 20% acetonitrile/water containing 0.5% formic acid and 25 ng of each internal standard, d_3_-quinolinate (Medical Isotopes, Inc., Pelham, NH, USA) and d_5_-tryptophan (Santa Cruz Biotechnology, Inc., Dallas, TX, USA). The sample was mixed by vortexing and was then centrifuged at 21,000× *g,* and the supernatant was collected to prevent any precipitate from being introduced into the UPLC column. Quinolinate and tryptophan were resolved by UPLC (40 °C) on a Acquity™ Premier HSS T3 (1.8 µm × 2.1 mm × 50 mm) column (Waters Corporation, Milford, MA, USA) using a solvents gradient starting from 0.6% formic acid in H_2_O to 0.6% formic acid in 90% CH_3_CN/H_2_O over 2.5 min at a flow rate 0.200 mL/min. Ions were monitored using multiple reaction monitoring in the positive ion mode: *m/z* 168.1 → *m/z* 77.91 and *m/z* 171.1 → *m/z* 80.91 for quinolinate and d_3_-quinolinate, respectively, and *m/z* 205.3 → *m/z* 188.1 and *m/z* 210.3 → *m/z* 192.1 for tryptophan and d_5_-tryptophan, respectively. Isotope ratios were used for quantification. The coefficient of variation for quinolinate measurements performed in standard quality control samples was 4.73%.

### 2.4. Statistical Analysis

Power calculations for this study were based on a previous study of urinary quinolinate and quinolinate to tryptophan ratios in patients undergoing on-pump cardiac surgery [11]. Due to the very large effect size and relative precision of the quinolinic acid assay, in cardiac surgery patients a significant difference in urinary quinolinate to tryptophan ratios was detected with a matched case-control design of six patients who developed postoperative AKI and six patients who did not develop AKI. In the previous study, the response within each subject group was normally distributed, with a standard deviation of 2. For this study, it was calculated that if the true difference in the AKI and non-AKI group means is 4, we would need to study six vascular surgery patients who developed AKI and six matched patients who did not develop AKI to be able to reject the null hypothesis that the population means of the AKI and non-AKI groups are equal, with a probability (power) of 0.9. The Type I error probability associated with the test of this null hypothesis was 0.05. For this study, eight patients who developed postoperative AKI were matched with eight patients who did not develop AKI to account for the possibility of a slightly lower effect size in vascular surgery patients compared to on-pump cardiac surgery patients.

For patient variables, demographic and clinical data were summarized as *n* (%) for binary variables and the 50th (10th, 90th) percentile for continuous variables. Two-tailed Student’s *t*-tests were used to compare continuous demographic and clinical variables and chi-squared tests were used to compare binary variables between AKI and non-AKI groups. Pearson’s correlation coefficients were used to examine the correlation between preoperative and postoperative renal function and urine quinolinate and quinolinate to tryptophan ratios. Two-sided Mann–Whitney U tests were used to compare urine quinolinate and quinolinate to tryptophan ratios between patients that did and did not develop AKI. Multivariate linear regression modeling was used to estimate the relationship between urine quinolinate and quinolinate to tryptophan ratios and the maximum change in serum creatinine from baseline 48 h after surgery. All model covariates were selected a priori. The models were adjusted for age, sex, baseline estimated glomerular filtration rate, and diabetes using the first two principle components of these factors in order to avoid model overfitting [27].

## 3. Results

### 3.1. Cohort Characteristics

Baseline characteristics of the preoperative cohort are shown in Table 1. All eight (100%) patients had hypertension in the group that developed postoperative AKI and in the group that did not develop postoperative AKI. Both groups of patients had two (25%) patients with type II diabetes. Per the enrollment criteria, all study patients had a preoperative eGFR less than 60 mL/min/1.73 m^2^. The median eGFR was 47 mL/min/1.73 m^2^ (10th percentile: 34 mL/min/1.73 m^2^, 90th percentile: 56 mL/min/1.73 m^2^) in the group of patients that developed postoperative AKI and 49 mL/min/1.73 m^2^ (41, 58) in the group of patients that did not develop postoperative AKI. In both the AKI and non-AKI groups, six (75%) of the patients underwent aortic aneurysm repair, three (38%) of which were endovascular. The remaining two patients in the AKI group underwent a femoral artery endarterectomy and a mesenteric artery bypass. In the non-AKI group, the remaining two procedures were both femoral–femoral bypass revisions. There was no statistical difference between the volume of iodinated contrast used in the surgical procedures of the patients who developed postoperative AKI compared to the volume of iodinated contrast used in the surgical procedures of the patients who did not develop postoperative AKI.

Baseline characteristics of the postoperative cohort are shown in Table 2. Similar to the preoperative cohort, all eight (100%) patients had hypertension in both the group that developed postoperative AKI and the group that did not develop postoperative AKI, and two (25%) patients in each group had type II diabetes. The median eGFR was 42 mL/min/1.73 m^2^ (29, 56) in the group that developed postoperative AKI and 47 mL/min/1.73 m^2^ (38, 58) in the group that did not develop AKI. Six (75%) patients in both the AKI and non-AKI groups underwent aortic aneurysm repair, four (50%) of which were endovascular. The remaining two (25%) procedures in the AKI group were a mesenteric artery bypass and a femoral artery endarterectomy, and in the non-AKI group they were both femoral–femoral bypass revisions. Similar to the preoperative cohort, there was no statistical difference between the volume of iodinated contrast used in the surgical procedures of the patients who developed postoperative AKI compared to the volume of iodinated contrast used in the surgical procedures of the patients who did not develop postoperative AKI.

### 3.2. Quinolinate Measurements

The median preoperative urinary quinolinate concentration was 12.621 μg/mL (7.291, 22.081) in the group that developed postoperative AKI and 7.019 μg/mL (3.604, 15.928) in the group that did not develop postoperative AKI. The median postoperative urinary quinolinate concentration was 9.349 μg/mL (5.470, 17.355) in the group that developed postoperative AKI and 8.200 μg/mL (3.658, 15.157) in the group that did not develop postoperative AKI. Neither preoperative nor postoperative urinary quinolinate concentration was correlated with preoperative eGFR (R = 0.07 and 0.08, *p* = 0.77 and 0.75, respectively).

**Figure 1:**
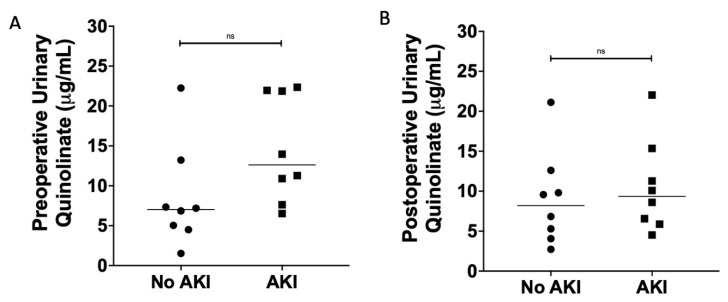
Urinary quinolinate concentrations for patients with and without postoperative AKI: (**A**) preoperative quinolinate concentration and (**B**) postoperative quinolinate concentration. ns denotes non-significant difference.

There was no difference in the preoperative urinary quinolinate concentrations between patients that did and did not develop postoperative AKI (*p* = 0.07, Figure 1A). Similarly, there was no difference in the postoperative urinary quinolinate concentrations between patients that did and did not develop postoperative AKI (*p* = 0.50, Figure 1B).

### 3.3. Quinolinate to Tryptophan Ratios

The median preoperative urinary quinolinate/tryptophan ratio was 0.840 (0.563, 1.613) in the group that developed postoperative AKI and 0.606 (0.405, 1.486) in the group that did not develop postoperative AKI. The mean postoperative urinary quinolinate/tryptophan ratio was 1.119 (0.434, 1.877) in the group that developed postoperative AKI and 0.380 (0.294, 0.705) in the group that did not develop postoperative AKI. Neither the preoperative nor postoperative urinary quinolinate/tryptophan ratio was correlated with preoperative eGFR (R = −0.36 and −0.27, *p* = 0.14 and 0.28, respectively).

**Figure 2:**
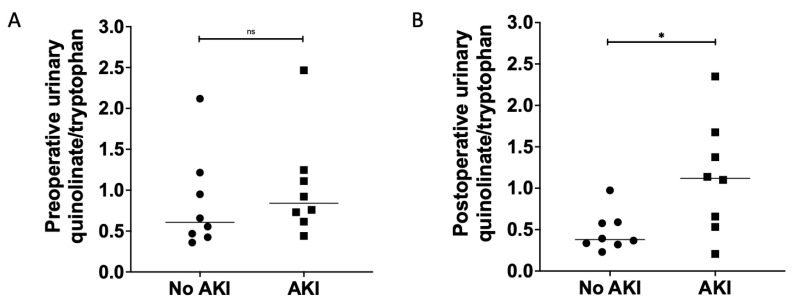
Urinary quinolinate/tryptophan ratio for patients with and without postoperative AKI: (**A**) preoperative quinolinate/tryptophan ratio and (**B**) postoperative quinolinate/tryptophan ratio. ns denotes non-significant difference, * denotes *p* < 0.05.

There was no difference in preoperative urinary quinolinate/tryptophan ratios between the AKI and non-AKI groups (*p* = 0.32, Figure 2A). However, there was a difference in the quinolinate to tryptophan ratio on the morning of postoperative day one between patients that did and did not develop postoperative AKI (*p* = 0.04, Figure 2B).

### 3.4. Association between Quinolinate and Postoperative Creatinine

**Figure 3:**
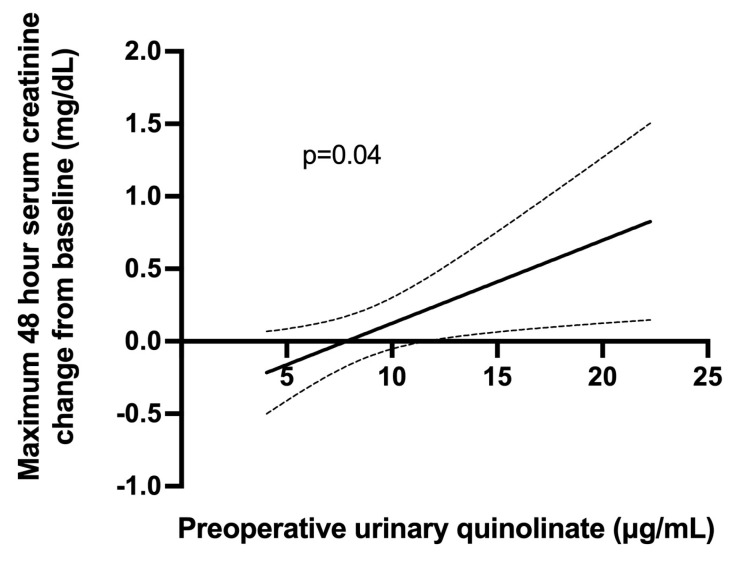
Preoperative urinary quinolinate concentrations and maximum change in serum creatinine from baseline 48 h postoperatively. Dotted lines denote the 95% confidence interval.

**Figure 4:**
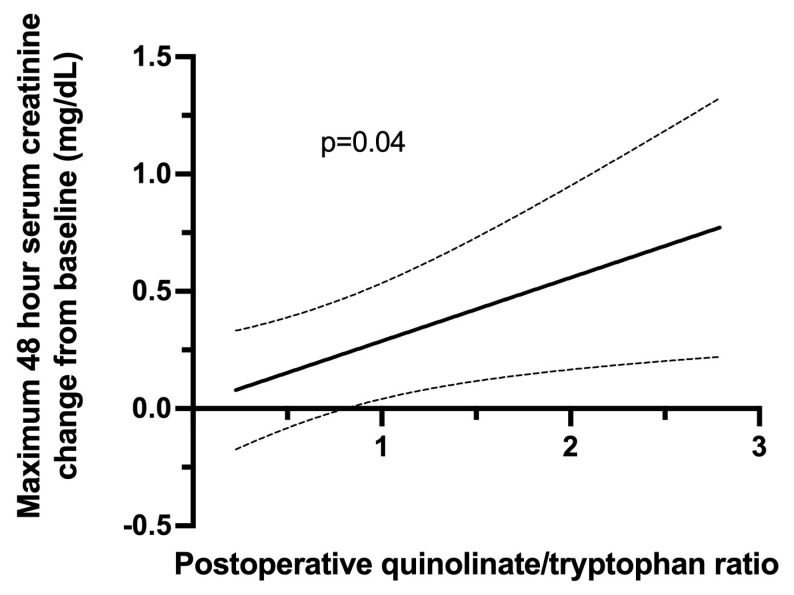
Postoperative urinary quinolinate/tryptophan ratio and maximum change in serum creatinine from baseline postoperatively. Dotted lines denote the 95% confidence interval.

The mean 48 h postoperative serum creatinine change from baseline was 0.71 mg/dL (0.27, 1.86) in patients that developed postoperative AKI and −0.13 mg/dL (−0.26, 0.04) in patients that did not develop postoperative AKI. The postoperative quinolinate/tryptophan ratio was positively correlated with the 24 h postoperative increase in serum creatinine from baseline (R = 0.58, *p* = 0.02) and with the 48 h postoperative increase in serum creatinine from baseline (R = 0.60, *p* = 0.02). After adjusting for age, sex, preoperative eGFR, and diabetes using principal component analysis, higher preoperative urine quinolinate concentrations were associated with higher 48 h maximum serum creatinine changes from baseline after surgery (*p* = 0.04, Figure 3). Additionally, higher urinary quinolinate to tryptophan ratios measured on the morning of postoperative day one were associated with higher 48 h maximum serum creatinine changes from baseline after surgery (*p* = 0.04, Figure 4).

## 4. Discussion

AKI is a common, costly, and serious adverse outcome after major vascular surgery. Postoperative AKI is independently associated with an increasing risk of chronic kidney dysfunction and both short- and long-term death [1,2,3,4,5,6]. It is estimated that the cost of in-hospital AKI supportive care is over five billion dollars annually in the United States and accounts for 1% of the entire National Health Service budget in the United Kingdom. Up to now, no effective preventative methods and treatments for AKI after vascular surgery have been identified, and the need for novel therapeutic targets is great. Elevated urinary quinolinate is a marker of reduced renal QPRT activity, an essential enzyme in the de novo synthesis of NAD+ from tryptophan. Reduced renal QPRT has been demonstrated in mouse models of AKI [11]. Recent clinical studies have also reported associations between urinary quinolinate concentration and the risk of developing AKI in critically ill patients, patients hospitalized with the COVID-19 virus, and patients undergoing cardiac surgery with exposure to cardiopulmonary bypass [11,14,17].

We demonstrated this association in adult patients with chronic kidney disease undergoing major elective vascular surgery. In this patient population, our data demonstrate that higher preoperative urinary quinolinate concentrations are independently associated with greater postoperative increases in serum creatinine concentration. Since all the patients in this study were undergoing elective, non-emergent surgery, this suggests that baseline inter-patient differences in NAD+ synthesis capacity may play an important and currently unrecognized role in the development of AKI after vascular surgery. Considering that urine samples can be readily and safely obtained from patients in preoperative clinics, in the in-patient hospital setting, and in presurgical preparation areas (the holding room), further assessment of urinary quinolinate concentration as an independent predictor of postoperative AKI in vascular surgery patients is warranted. Preoperative detection of patients with an impaired NAD+ synthesis capacity through urinary quinolinate concentration measurement could alert clinicians to the need for AKI risk reduction strategies in these patients, such as minimizing exposure to intravenous contrast compounds and nephrotoxic medications and increasing vigilance and monitoring for the development of AKI throughout the perioperative period. Further, considering that all the factors included in our multivariate analysis can be measured or ascertained before surgery, our data support the possibility that preoperative urinary quinolinate concentration might be incorporated into a preoperative predictive tool for improved identification of vascular surgery patients at high risk of developing postoperative AKI, allowing clinicians to target interventions to these high-risk patients. Future work should be performed to develop this AKI predictive algorithm, to compare its predictive performance to current AKI predictive models, and if it shows improved predictive ability, to validate it in this patient population.

Similarly, in this study we found that in patients undergoing major vascular surgery, higher urinary quinolinate to tryptophan ratios on the morning of postoperative day one were associated with greater postoperative renal injury. Since AKI is often not detectable by serum creatinine changes until 48 h or more after renal injury occurs, this suggests that urinary quinolinate to tryptophan ratios might be developed as a biomarker which could more rapidly detect postoperative AKI in vascular surgery patients than current methods. Further, studies have shown that higher urinary quinolinate to tryptophan ratios after deceased-donor kidney transplantation independently predicts reduce allograft function 3 to 12 months after transplantation and the risk of progression to chronic kidney disease in the graft recipient [14]. It is possible that postoperative urinary quinolinate to tryptophan ratios in vascular surgery patients may also be associated with long-term renal functional decline. Futures studies are warranted to investigate this relationship.

Importantly, treatments are under development and clinical testing that might protect kidney function in surgical patients with inherent or acquired NAD+ synthesis deficiencies. One of these treatments is NAM. High doses of NAM are well-tolerated by patients and have been administered in clinical trial settings for up to five years without adverse effects. In a recent phase I trial, oral NAM increased whole blood NAD+ levels in hospitalized patients with AKI and was safe in doses of up to 1000 mg twice daily [28]. Further, a small clinical trial (NCT03727646) for oral nicotinamide riboside in patients with heart failure demonstrated that 5–9 days of treatment reduced PBMC cytokine production, including IL-6, suggesting that nicotinamide-based treatments can rapidly reduce systemic inflammation, a known risk factor for postoperative AKI [29]. Currently, two phase 2 clinical trials are underway to evaluate perioperative NAM supplementation for prevention and treatment of renal injury in on-pump cardiac surgery patients (NACAM: NAD+ Augmentation in Cardiac Surgery-Associated Myocardial Injury Trial, change in eGFR is a secondary trial outcome, NCT04750616), and in patients undergoing complex aortic aneurysm repair and open aortic arch reconstruction (Protection From Acute Kidney Injury (AKI) With Basis Treatment, NCT04342975). A third ongoing trial (VIBAKI: Intravenous Administration of Vitamin B Complex Improves Renal Recovery in Patients with AKI, NCT04893733) is examining the effect of niacin supplementation on AKI development and recovery in both surgical and non-surgical patients. Results from these trials will help researchers interested in developing AKI preventive treatments for various vascular surgery patient populations better determine the possible benefit of perioperative NAM and niacin supplementation for AKI prevention in these patient populations.

The small sample size utilized in this study is its most notable limitation. Our sample size was limited due to the expensive nature of the UPLC-MS/MS measurement technique we used and does limit our ability to extrapolate these data to a wide variety of patient populations. However, the patient cohorts used in this study were well-matched to reduce bias and the study was strongly powered to detect our outcome of interest. Further, in addition to univariate analysis used in the previous study of cardiac surgery patients, we also performed multivariate analysis with principal components’ adjustment to remove the influence of as many confounders as possible from our assessment of urinary quinolinate concentration and urinary quinolinate to tryptophan ratios in vascular surgery patients that do and do not develop postoperative AKI. This multivariable analysis further improved the statistical power to detect differences in these laboratory values and reduces residual bias that may remain due to residual differences in the matched cohorts. In conclusion, our data demonstrated that elevated urinary quinolinate and quinolinate to tryptophan ratios, markers of reduced QPRT activity, and impaired NAD+ synthesis from tryptophan were independently associated with the development of postoperative AKI in a small case-control study of adult patients undergoing major elective vascular surgery. Future studies confirming these findings in larger cohorts of patients are needed. Further, additional studies examining the relationship between perioperative urinary quinolinate concentration and quinolinate to tryptophan ratios and the risk of AKI, with a focus on specific vascular surgery patient populations, such as patients undergoing endovascular aortic repair and patients undergoing open vascular repair, need to be performed to determine if specific risk factors for AKI, such as exposure to high doses of iodinated contrast or aortic clamp time (ischemic time), are associated with QPRT activity and impaired NAD+ synthesis from tryptophan.

Additionally, the existing mass spectroscopy-based assay is expensive, not readily available at every institution, and therefore prohibitive as a large-scale measurement method for urinary quinolinate and quinolinate to tryptophan ratios. Therefore, the development of a more affordable, accessible, high-throughput assay for quinolinate and tryptophan quantification in urine would advance this field of study and make it possible to study NAD+ synthesis impairment in large cohorts of surgical patients.

## Figures and Tables

**Table 1:** Preoperative cohort characteristics.

Characteristic *	AKI (*n* = 8)	No AKI (*n* = 8)	*p*-Value
Age	70 (55, 79)	73 (58, 83)	0.58
Female	4 (50%)	3 (38%)	0.61
Body mass index (kg/m^2^)	27 (34, 35)	30 (23, 37)	0.32
Medical history			
Hypertension	8 (100%)	8 (100%)	1
Type II diabetes	2 (25%)	2 (25%)	1
eGFR (mL/min/1.73 m^2^)	47 (34, 56)	49 (41, 58)	0.36
Procedure characteristics			
IV contrast use	3 (38%)	3 (38%)	1
Volume of IV contrast (mL)	0 (0, 132)	0 (0, 122)	0.72

* Continuous variables are reported as the 50th (10th, 90th) percentile, and binary variables are reported as *n* (%). eGFR: estimated glomerular filtration rate.

**Table 2:** Postoperative cohort characteristics.

Characteristic *	AKI (*n* = 8)	No AKI (*n* = 8)	*p*-Value
Age	72 (65, 79)	74 (66, 83)	0.58
Female	4 (50%)	2 (25%)	0.30
Body mass index (kg/m^2^)	26 (23, 31)	29 (23, 34)	0.31
Medical history			
Hypertension	8 (100%)	8 (100%)	1
Type II diabetes	2 (25%)	2 (25%)	1
eGFR (mL/min/1.73 m^2^)	42 (29, 56)	47 (38, 58)	0.14
Procedure characteristics			
IV contrast use	4 (50%)	4 (50%)	1
Volume of IV contrast (mL)	18 (0, 132)	0 (0, 122)	0.72

* Continuous variables are reported as the 50th (10th, 90th) percentile, and binary variables are reported as *n* (%). eGFR: estimated glomerular filtration rate.

## Data Availability

The data presented in this study are available on request from the corresponding author. The data are not publicly available due to study consent constraints with the goal of protecting patient privacy.
